# Environment matters: How are neighborhood structural indexes associated with parenting stress among Asian immigrant families?

**DOI:** 10.1371/journal.pone.0293594

**Published:** 2023-11-29

**Authors:** Fei Pei, Susan Yoon, Fuhua Zhai, Qin Gao

**Affiliations:** 1 David B. Falk College of Sport and Human Dynamics, School of Social Work, Syracuse University, Syracuse, NY, United States of America; 2 College of Social Work, The Ohio State University, Columbus, OH, United States of America; 3 Graduate School of Social Service, Fordham University, Bronx, New York, United States of America; 4 School of Social Work, Columbia University, New York, New York, United States of America; 5 Department of Social Welfare, College of Social Sciences, Ewha Womans University, Seoul, South Korea; St John’s University, UNITED STATES

## Abstract

The Asian immigrant population is the fourth largest immigrant population in the United States, and its parenting stress issues have been consistently recognized in previous studies. However, little attention has been paid to neighborhood-level factors and their parenting stress. Using the Study of Asian American Families and 2016 American Community Survey 5-year estimates, this study examined the association between neighborhood structural indexes and Asian immigrant parents’ parenting stress, along with the mechanism driving the relationship. We found that cultural orientation and social support fully mediated the effects of economic disadvantages on parenting stress among Asian immigrant parents. Only cultural orientation mediated the direct effects of ethnic heterogeneity on Asian parents’ parenting stress. Improving Asian immigrants’ living environment, including economic status and ethnic diversity, would be critical to relieve the parenting stress of Asian immigrant families. Interventions and preventions to increase social support, and inform cultural orientation and acculturation are emphasized.

## Introduction

Parenting is vital for healthy child development, yet parenting stress, defined as the experiences and reactions that parents encounter when trying to manage the challenges and difficulties of parenthood [1], can increase family dysfunction and bring mental health issues among both parents and children. Racial and ethnic minorities, including Asian immigrant parents, experience higher levels of parenting stress than White parents due to structural disadvantages, lack of social support and economic resources, discrimination, and cultural differences [2]. Although Asian immigrants represent one of the most rapidly growing minority populations in the United States and have higher levels of parenting stress compared to other racial and ethnic groups [3,4], little attention has been paid to their parenting stress and its implications for child development.

A growing body of research underscores the important role of broader community and neighborhood contexts in parenting stress [5–7], yet the impact of neighborhood factors on parenting stress has not been rigorously examined among Asian immigrant parents. Furthermore, the underlying mechanisms through which neighborhood structural factors influence parenting stress in this population remain unclear. Understanding how environmental contexts (e.g., neighborhood structures) may contribute to parenting stress and examining cultural and social contexts (e.g., cultural values, cultural practices, social support) as potentially modifiable intervention targets is essential to devising effective intervention strategies to reduce parenting stress and promote positive child and family outcomes among Asian immigrant families. In this study, we sought to understand the associations between neighborhood structural indexes (i.e., economic disadvantage, ethnic heterogeneity) and parenting stress among Asian American immigrant families and whether social support, cultural values, and cultural practices mediate these associations.

### Parenting stress among asian immigrant families

Parenting stress refers to experiences and reactions that parents encounter when trying to manage the challenges and difficulties of parenthood [1]. The stress of parenthood is distinct from other types of life stresses due to its consistent, daily nature. Parenting stress is prevalent worldwide, and persistently high levels of parenting stress have substantial individual, familial, and societal consequences, including increased risk of maternal mental illness, child maltreatment, and poor child development due to trauma [8,9]. Moreover, the Asian immigrant population in the United States has been growing rapidly and its parenting stress is significantly higher compared to other racial groups in previous empirical articles [3,4,10]. However, these immigrants’ parenting behaviors and needs have not been well understood and services are severely lacking. From a culturally informed perspective, parenting stresses are distributed unequally across ethnic groups due to structural disadvantages and different parenting values [11]. Asian immigrants in this study include immigrants from East Asia, South Asia, and Southeast Asia. Although these subgroups of Asia have different cultural heritages, there are similarities in their cultural values [4,12]. Compared with American culture, traditional Asian cultures emphasize children’s respect for elders, obedience to parents, and parents’ self-sacrifice to enhance child well-being [13]. These differences in cultural values and beliefs bring additional stress to Asian immigrant parents [10]. Nomaguchi and House [14] suggested that because of limited resources in their new country, lack of social support, and different parenting values, Asian parents reported higher stress levels than White parents.

A substantial body of research has investigated risk and protective factors related to parenting stress among immigrant families of different ethnicities [10,15]. Besides commonly discussed factors including family income, education levels of parents, and children’s developmental problems [16,17], perceived insufficient support from family and friends, dysfunctional family ecology, and acculturation stress are additional factors that increase immigrants’ parenting stress [10,15,18]. For example, using a nationally representative dataset of more than 1,000 families, Driver and Shafeek Amin [18] found that both acculturation and social support influenced maternal parenting stress. However, most previous studies examined individual- and family-level predictive factors of parenting stress, and fewer studies investigated the influences of living environment (neighborhood factors). Thus, efforts to prevent and ameliorate parenting stress are needed outside of a traditional individual-level focus via an increased understanding of neighborhood contextual factors that influence parenting stress.

### Living environment matters: Neighborhood structural indexes

Using Bronfenbrenner’s [19] ecological theory, predictive factors of parenting stress can be categorized as individual-, family-, and neighborhood-level factors. Research on neighborhood-level factors and parenting stress yielded inconsistent findings [5,20,21]. For example, McCloskey and Pei [20] suggested that neighborhood social cohesion had direct positive effects on maternal parenting stress; Wang et al. [22] had similar findings that both neighborhood collective efficacy (positively) and poverty (negatively) affected parenting stress. Franco et al. [5] found that neighborhood disorder was positively associated with parenting stress using the same dataset, but minority parents showed less stress than White parents in disadvantaged neighborhoods. Therefore, further investigations of the influences of neighborhood factors on parenting stress are warranted.

Moreover, given various types of neighborhood factors, a clear definition is needed before further discussion of their influences. Three major types of neighborhood factors have been identified in the previous literature: neighborhood collective efficacy (including social cohesion and social control), neighborhood disorder, and neighborhood structural factors (i.e., neighborhood characteristics; [23]). Neighborhood collective efficacy refers to the relationship among residents and their willingness to support one another [24]. Neighborhood disorder mainly involves violent activities and problems of a certain geographic area [24]. Finally, neighborhood structural factors are indexes that reflect the economic, residential status, and ethnicity of a neighborhood, such as the percentage of households with income below the poverty line and the percentage of minorities living in a neighborhood [25]. Previous studies focused on investigating the influences of various neighborhood factors on child maltreatment, substance use, and mental disorders, but few studies captured influences of neighborhood factors on immigrants, who had significantly different living environments compared to other populations. In particular, neighborhood structural factors, as the most commonly discussed neighborhood factors, have not been discussed regarding Asian immigrants’ parenting.

According to social disorganization theory [25], neighborhood structural indexes can potentially affect individuals’ behaviors, including parenting styles and stress. Neighborhood economic disadvantage is commonly considered to negatively affect child development [26,27]. Additionally, immigrants tend to be segregated in certain neighborhoods, and their environment has unique influences on their daily life and behaviors. For example, the minority stress framework suggests living in neighborhoods with a high proportion of Asian Americans will decrease Asian immigrants’ stress level [28,29]. However, little is known about the influences of their living environment. Few studies discussed the relationship between neighborhood structural indexes and Asian immigrant children’s behavior problems and child maltreatment [21,30]. Thus, further scientific knowledge about the underlying mechanisms through which neighborhood structural indexes influence parenting stress in this group is needed. Investigating parenting issues among Asian American families from a neighborhood perspective will fill a critical gap in the knowledge of minority families’ well-being as individuals’ behaviors are affected by their living environment but limited studies investigate this issue among minority families.

### Two mediators: Cultural orientation and social support among immigrants

The United States has a large immigrant population: Nearly 1 in 4 children are from immigrant families in 2019 [31]. For immigrant families, their adventure in a new country is accompanied by changes across life domains with both challenges and opportunities, especially with respect to cultural beliefs and identifications [32]. The immigrant process differs between individuals and those of different national origins. However, a common challenge for immigrants is dealing with the adjustment between their original and host cultures. Particularly, Asian cultural values and heritages are significantly different from Western culture in the United States [33]. Although Asian cultures include cultures in East Asia, South Asia, and Southeast Asia, there are significant similarities across these cultural values [12]. Mainly influenced by Confucianism, Buddhism, and Hinduism, the Asian parenting style (including various subcultures like South Asian, East Asian, and Southeast Asian) emphasizes prioritizing family, respecting and obeying parental authority, and ensuring children’s education [34,35].

Immigrants’ cultural orientation process has been well investigated in the past decades. Cultural orientation refers to how individuals endorse, engage in, and practice one or more cultures, traditions, norms, and beliefs [33,36,37]. According to the bilinear cultural adaptation model [38], there are two types of cultural orientations: acculturation and enculturation. Acculturation describes the process of immigrants adapting to their mainstream host culture. Enculturation emphasizes the process of immigrants maintaining or preserving their original culture [39–44]. Early-stage investigation of acculturation and enculturation found a unidimensional model of incompatibility [45], meaning adaptation to a host culture is irreversible and affects the heritage of immigrants’ original culture. Recently, as more studies have paid attention to bicultural families, researchers have suggested that acculturation and enculturation processes can happen simultaneously [42,46]. In the current study, a bidimensional concept is adopted, with cultural orientation assessed based on both acculturation and enculturation.

The relationship between neighborhood ethnic heterogeneity and Asian immigrants’ cultural identification is well established [28,29,47]. For example, in a study with 1,583 immigrant Asians, Leu et al. [47] suggested that living with more Asian Americans increases their preference for traditional Asian cultures, which is consistent with previous work [42,48]. Also, such preference is thought to be related to lower stress levels due to acculturation. However, the influences of neighborhood economic status and residential instability on Asian immigrants’ cultural preferences have not been commonly discussed in previous research.

Previous studies suggested that cultural orientation is connected to parenting stress level among immigrant families [33,49]. The conflicts between their traditional culture and the culture of the new country bring additional stress for immigrant families. The minority stress framework suggests that an individual’s minority status is related to their stress levels, which includes minority stressors like ethnic identity [50,51]. A study of 570 Latinx families found that acculturation stress increased maternal depression [49], a finding supported by a study of Chinese immigrant families [42]. Similarly, enculturation was associated with greater family life satisfaction among Arab immigrants in Canada [46]. Although few articles discussed the relationship between neighborhood factors and parenting stress, some studies noted the negative influences of neighborhood structural indexes and social cohesion on parenting stress [20–22]. Both low social cohesion and neighborhood economic disadvantages were shown to be positively related to parenting stress in previous research [22]. Therefore, understanding the mediation role of cultural orientation in the relationship between neighborhood structural indexes and parenting stress among Asian immigrant families will benefit the well-being of this historically underserved ethnic group.

Additionally, Barrera’s [52] social support theory posits that structural social support includes support from family, friends, and local groups, which may serve as a protective factor to buffer the negative influences of neighborhood indexes on individuals and decrease parenting stress. Such a mediating relationship was supported in a study of 1,045 families [7]: Social support mediated the negative relationship between neighborhood social cohesion and parenting stress. Similar findings were noted among 1,300 Korean families using moderating models [53]. However, to the authors’ knowledge, no study has comprehensively investigated relationships among neighborhood structural indexes, cultural orientation, social support, and parenting stress in Asian immigrant families.

### The present study

**Fig 1:**
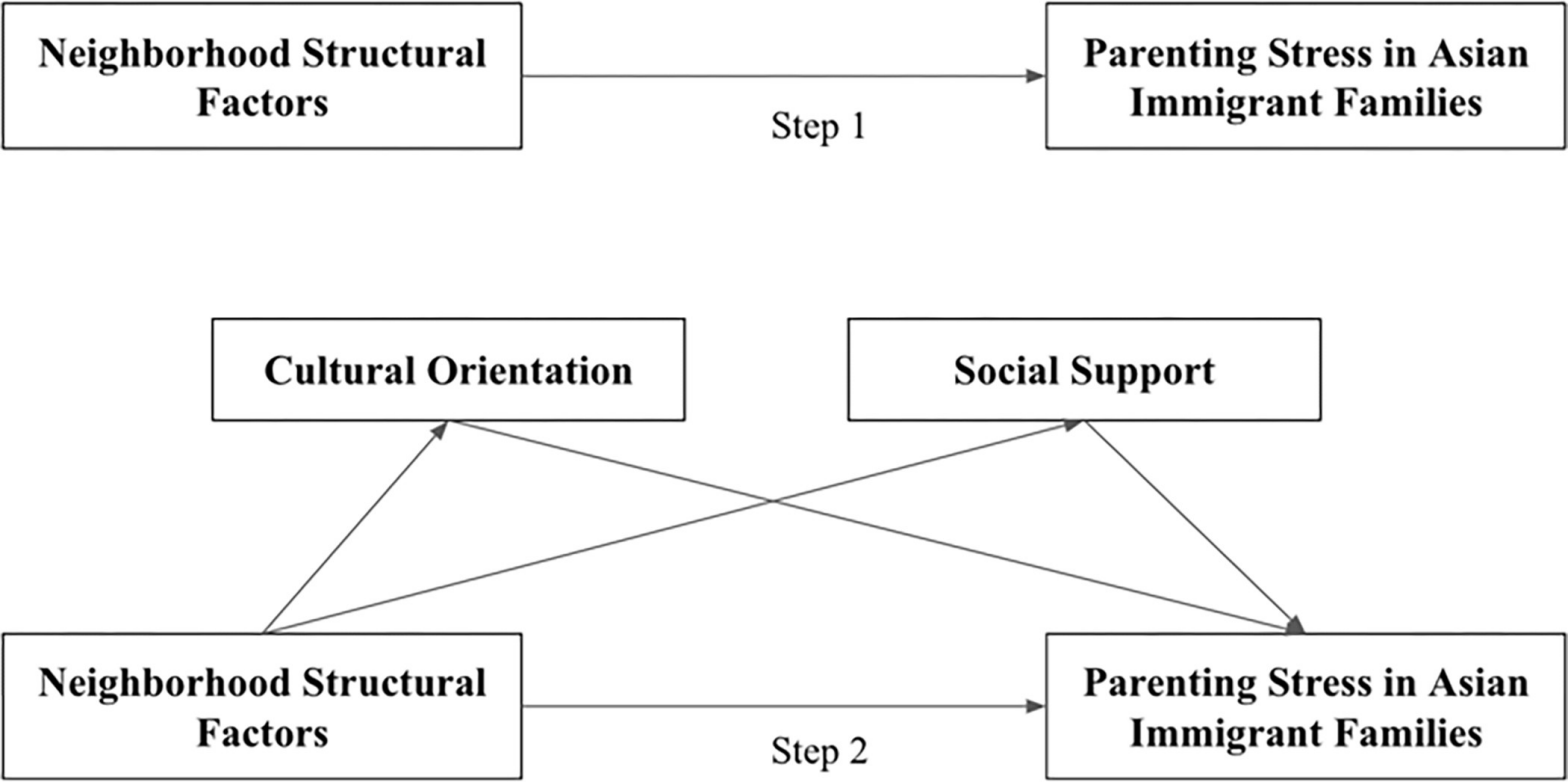
Conceptual model.

To gain insights to inform the development of supportive communities for Asian immigrant families and promote ethnic equity by elucidating powerful, modifiable contextual indexes linked to parenting stress, this study addressed two questions: (1) Is there a direct relationship between neighborhood structural indexes and parenting stress among Asian immigrant families? And (2) Do cultural orientation and social support indirectly affect parenting stress among Asian immigrant families? (See Fig 1).

## Method

This study used data from two sources. First, the Study of Asian American Families (SAAF) includes information about Asian American children and parents’ cultural values and practices, parenting behaviors, and social behaviors. SAAF included 964 Asian children aged 0–18 and their parents in New York (2011–2012), New Jersey (2013–2014), and Hawaii (2015–2017), three states in which Asian Americans are concentrated. The inclusion criteria for the SAAF study were those who self-identified as an Asian parent with one or more children under the age of 18 living in the same household. Finally, this study includes immigrants mainly (79.83%) from China, Japan, South Korea, India, and others (including Asian Pacific Islanders, and parents from other Asian countries). The second data source was the 2016 American Community Survey 5-year estimates, which contain information about the neighborhood environment at the ZIP code level. Neighborhood structural indexes, such as percentages of families below the federal poverty line, renter-occupied homes, and families receiving public assistance, were pulled from this dataset. The two datasets were matched using ZIP codes, and full maximum likelihood estimation was applied to address missing data. The final sample size was 849 after excluding 115 participants lacking data for all key variables.

### Measures

#### Parenting stress

Parenting stress was a latent variable measured by 12 items from the Parenting Stress Index Short Form [54]. These items captured parental distress levels and how parents adjusted their life. Items included “I often have the feeling that I cannot handle things very well” and “Since having a child, I feel that I am almost never able to do things that I like to do.” Parents answered these 12 questions on a 5-point scale (*strongly disagree* to *strongly agree*). Higher scores indicated higher parenting stress, and Cronbach’s alpha was .90.

#### Neighborhood structural indexes

Neighborhood structural indexes included three indexes: neighborhood economic disadvantages, residential instability, and ethnic heterogeneity [25,55]. Neighborhood structural indexes were measured by seven items from the 2016 American Community Survey 5-year estimates. Due to the high correlation among these items, principal component analysis was applied to construct these three indexes following previous empirical studies [56,57]. Economic disadvantage was constructed by four items for each ZIP code: percentage of civilian labor force members (aged 16 or older) who were unemployed (*M* = 3.90, *SD* = 1.26), percentage of families below the federal poverty line (*M* = 10.57, *SD* = 6.96), percentage of households on public assistance (*M* = 18.34, *SD* = 7.00), and percentage of female-headed households (*M* = 7.51, *SD* = 4.10). Residential instability reflects the frequency of moving in a specific area. The percentage of renter-occupied homes (*M* = 50.97, *SD* = 20.96) and percentage of families who moved within 1 year (*M* = 3.93, *SD* = 1.63) were used to measure residential instability. Ethnic heterogeneity captures the different ethnicities in a neighborhood (Castellini et al., 2011). Ethnic heterogeneity was indexed by percentage of non-Asian residents in a ZIP code (*M* = 65.15, *SD* = 19.89).

#### Cultural orientation

Cultural orientation was measured with two domains in the SAAF: cultural values and cultural practices. Two latent variables were constructed in this study. Cultural values were measured by 12 binary items (1 = *yes*, 0 = *no*) from the Multiphasic Assessment of Cultural Constructs-Short Form [58]. These 12 items captured parents’ attitudes toward familism, which reflects the cultural traditions of Asian Americans. Simple items included “More parents should teach their children to be loyal to the family” and “No matter what the cost, dealing with my relatives’ problems comes first.” Affirmative answers reflected a preference for their original cultural values, and internal consistency was .71. Another latent variable, cultural practices, was assessed by four items from the Korean Acculturation Scale [59]. These four items asked parents to rate their behaviors and preferences regarding spoken language and daily language use on a 5-point scale. To be consistent with cultural values, all four items were reverse coded, and higher scores reflected a preference for their original culture and language. Cronbach’s alpha was .80 in this study.

#### Social support

Social support was measured by the 7-point Multidimensional Scale of Perceived Social Support, consisting of 12 items. Questions ask about parents’ support from family, friends, and significant others, with items like “I can count on my friends when things go wrong,” “My family really tries to help me,” and “There is a special person with whom I can share my joys and sorrows.” Higher scores indicated stronger social support from others. The scale had good internal consistency (Cronbach’s alpha = .95).

#### Covariates

Demographic information, including parents’ gender, age, subethnicity (Chinese, Korean, Japanese, Filipinos, Indian, Bangladesh, Pakistanis, and Others), and highest education level (higher than college or not), children’s gender and age, and the number of children younger than 18 in the household, were controlled in the present study. Parents’ immigration status also was controlled in this study, measured by the number of years in the United States because most SAAF families (more than 94%) were first-generation immigrants.

### Data analysis

Mplus 8.0 was used to estimate structural equation models. To answer the two research questions, the direct relationship between neighborhood structural indexes and parenting stress among Asian immigrant families was examined first. Second, a mediation model was conducted to test the role of cultural orientation and social support in the relationship between neighborhood structural indexes and parenting stress. Bootstrapping was applied to test the indirect effects. Comparative fit index (CFI), root mean square error of approximation (RMSEA), and standardized root mean squared residual (SRMR) were used to assess model fit, and CFI greater than .95, RMSEA less than .05, and SRMR less than .08 indicated a good model fit [60].

## Results

Descriptive statistics of all variables are shown in Table 1; 78.1% of respondents were female and their average age was 45.7. The children’s average age was 14.49 at the time the parents were interviewed. Approximately half of the parents held bachelor’s degrees or above. The average number of years in the United States was 23.94. More than 60% of the participants lived in New York, and the neighborhood structural items are listed in Table 1.

**Table 1:** Descriptive statistics of study variables (N = 849).

	*M* (*SD*)	%	Range
Parent characteristics			
Gender (female)	663	78.1	
Age	45.75 (7.67)		24–80
Highest education			
Less than high school	125	14.7	
High school or Equal (less than college)	317	37.3	
College or above	407	48.0	
Asian Ethic Subgroup			
Chinese	357	42.0%	
Korean	143	16.8%	
Japanese	108	12.7%	
Filipinos	65	7.7%	
Indian	61	7.2%	
Bangladesh	25	2.9%	
Pakistanis	17	2.0%	
Others	73	8.7%	
Child characteristics			
Gender (female)	419	49.4	
Age	14.49 (5.14)		4–18
Children younger than 18 in household	1.87 (0.67)		1–6
Years in the United States (parents)	23.94 (8.29)		7–56
State			
New York		64.4	
New Jersey		11.9	
Hawaii		23.7	
Economic disadvantages (ZIP code)			
Unemployed (aged 16 or older)	3.9 (1.26)		9–12.60
Families below poverty line	10.57 (6.96)		0–37.30
Households on public assistance	18.34 (7.00)		3.83–56.99
Female-headed households	7.51 (4.10)		0–25.97
Residential instability (ZIP code)			
Renter-occupied housing	50.97 (20.96)		3.5–95.60
Moved within 1 year	3.93 (1.63)		0.50–11
Ethnic heterogeneity (non-Asian; ZIP code)	65.15 (19.89)		26.3–98.7

*Note*. Only observed variables are included in this table. Latent variables are not included in this table.

### Direct effects

**Fig 2:**
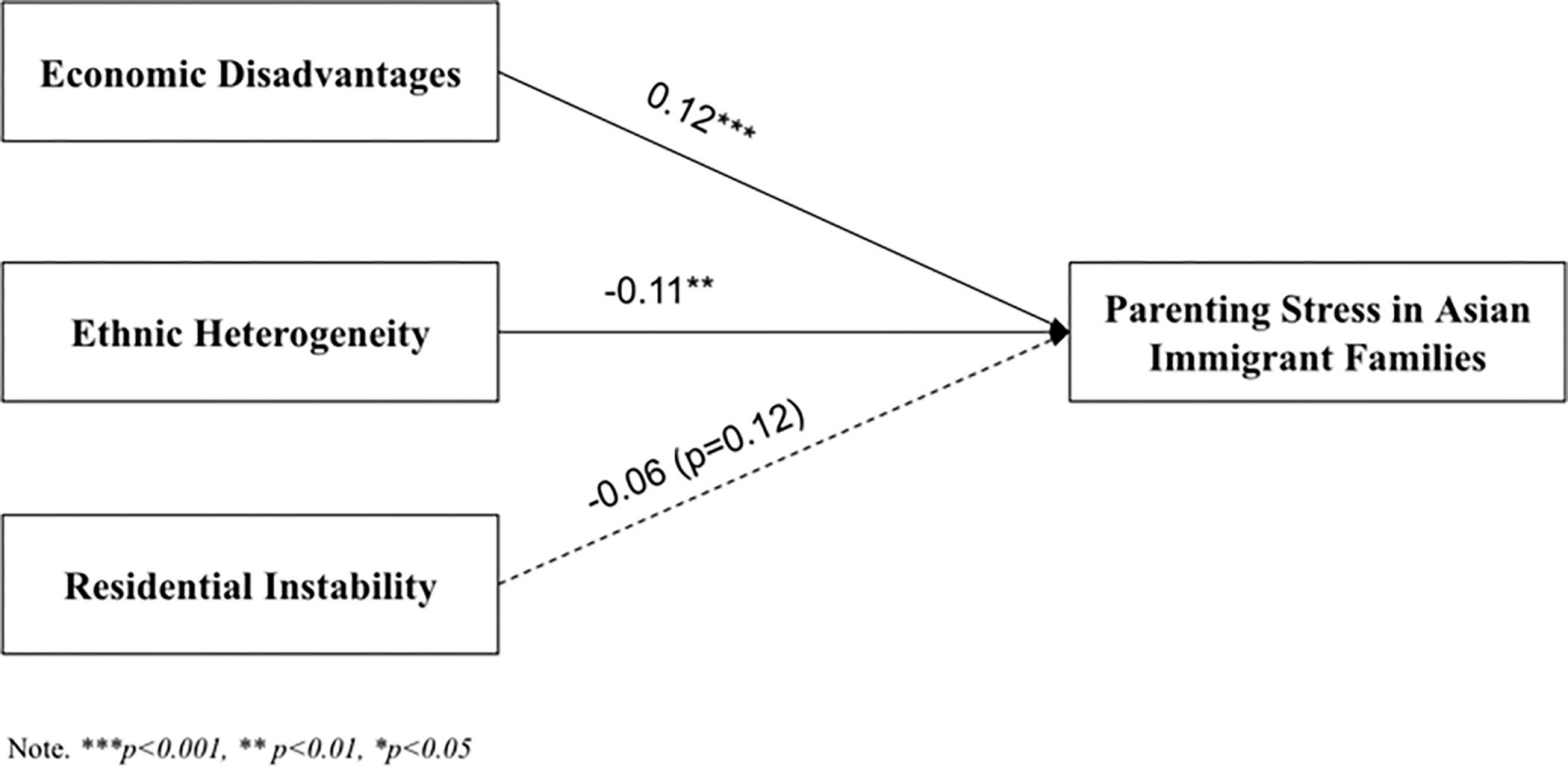
Direct effects. Note. ***p<0.*001*, **p<0.*01*, *p<0.*05*.

Fig 2 shows the total effects of three neighborhood structural indexes on parenting stress among Asian American families. The model fit was good for the initial model, CFI = .97, RMSEA = .06, SRMR = .049. Living in a neighborhood with high economic disadvantages was associated with higher parenting stress among Asian immigrant families (*b* = 0.12, *p* = .001). A higher percentage of non-Asian residents was related to lower parenting stress among Asian immigrant families (*b* = -0.11, *p* = .002). Residential instability did not have a significant relationship with Asian immigrant parents’ parenting stress (*b* = -0.06, *p* = .12).

### Mediating role of cultural orientation and social support

**Fig 3:**
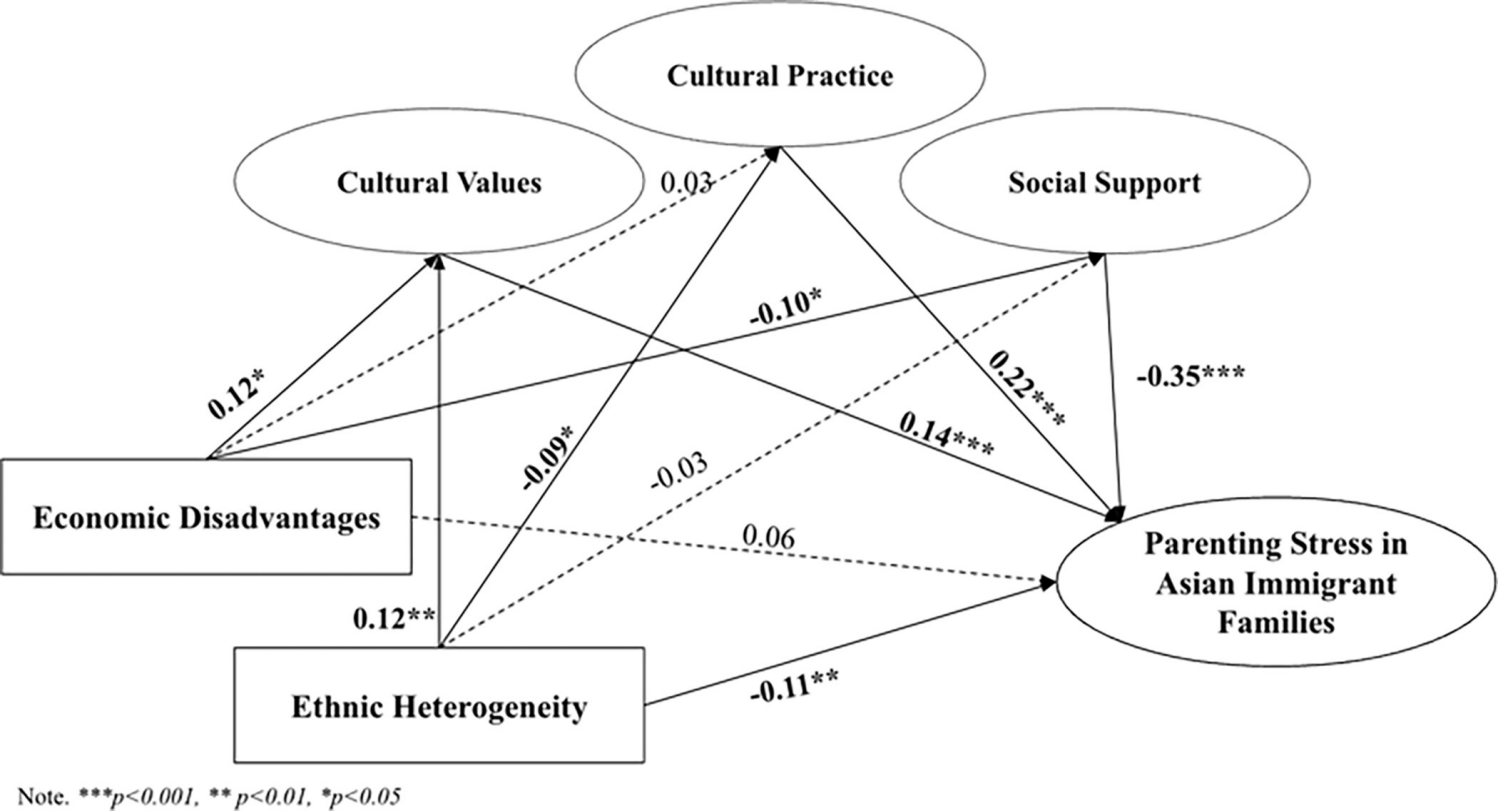
Mediation model. Note. ***p<0.*001*, **p<0.*01*, *p<0.*05*.

Fig 3 presents the results of analyses of the mediating role of cultural orientation and social support on the relationship between neighborhood structural indexes and parenting stress among Asian immigrant families. The mediation model yielded an acceptable model fit: CFI = .97, RMSEA = .048, SRMR = .09. As summarized in Table 2, the total indirect effect of neighborhood economic disadvantages on parenting stress was 0.06 (*p* = .02), and cultural values and social support significantly mediated the effects of economic disadvantages on parenting stress (indirect effect = 0.02 and 0.03, respectively). Asian parents living in a neighborhood with high economic disadvantages had stronger preferences for their traditional culture (*b* = 0.12, *p* = .01), which increased their parenting stress (*b* = 0.14, *p* < .001). Similarly, living in an economically disadvantaged neighborhood was related to lower social support (*b* = -0.10, *p* = 0.02), which led to higher parenting stress (*b* = -0.35, *p* < .001). The direct effect from economic disadvantage to parenting stress was not significant, and cultural practice did not play a mediating role in this process. Moreover, the direct effect from neighborhood economic disadvantage to parenting stress was no longer significant. This met one criterion for mediation in the causal steps approach, which states that the direct effect of X on Y becomes smaller or weaker after adding mediators, demonstrating that the mediator potentially plays a significant mediating role.

**Table 2:** Direct and indirect effects of neighborhood structure factors on parenting stress among Asian American parents.

	*b*	*SE*
Economic disadvantages to parenting stress (total)	0.11**	0.04
Direct effect	0.06	0.04
Indirect effect	0.06*	0.02
Via social support	0.03*	0.01
Via cultural values	0.02*	0.01
Via cultural practice	0.007	0.01
Ethnic heterogeneity to parenting stress (total)	-0.10**	0.04
Direct effect	-0.11**	0.03
Indirect effect	0.008	0.02
Via social support	0.012	0.01
Via cultural values	0.016*	0.008
Via cultural practice	-0.02*	0.01

*Note*. Standard errors are bootstrapped.

*p < .05.

**p < .01.

As for neighborhood ethnic heterogeneity (Table 2, Fig 3), the total indirect effect from ethnic heterogeneity to parenting stress among Asian immigrant parents was 0.008, which was not significant (*p* = .70). However, cultural values and cultural practices significantly mediated the relationship between ethnic heterogeneity and parenting stress among Asian immigrant parents (indirect effects = 0.016 and -0.02, *p* = .038 and .049, respectively). That is, living in a neighborhood with more residents from other ethnicities increased beliefs in traditional culture (*b* = 0.12, *p* = .006), and in turn, increased the parenting stress level (*b* = 0.14, *p* = .001). Asian immigrants living in a neighborhood with more individuals from other ethnicities embraced more behaviors from American culture (*b* = -0.09, *p* = .03), which also increased their parenting stress (*b* = 0.22, *p* < .001). There was a significant direct effect from ethnic heterogeneity to parenting stress (*b* = -0.11, *p* < .001), meaning that living in a neighborhood with more residents from other ethnicities was associated with lower parenting stress in these Asian immigrant families.

## Discussion

This study investigated the relationship between neighborhood structural indexes on parenting stress among Asian immigrant families and the mediating effects of cultural orientation and social support on this relationship. The findings contribute to the field in two ways. First, this study examined neighborhood-level community effects among Asian immigrants, which is an underserved population in terms of mental health and well-being. The Asian immigrant population has grown rapidly, but its parenting stress and behaviors and needs are not well understood, and related services are severely lacking. Asian immigrants usually live in specific neighborhoods, but little is known about the characteristics and impacts of these neighborhoods. Second, this study focused on the interactive effects of cultural orientation, social support, and neighborhood factors on minority families’ well-being, going beyond the traditional individual-level perspective. Investigating dynamic living environments and cultural differences creates a more realistic model that better captures the life changes among Asian immigrant families and their parenting stress.

### Direct effects

Consistent with previous findings in the general population [7,20], living in a neighborhood with high economic disadvantage was associated with higher parenting stress among Asian immigrant families. Neighborhood economic disadvantage is well documented as a major negative influence on residents’ psychological status and behaviors [26]. Living in a neighborhood with high economic disadvantage can bring inconvenience to residents’ daily life and increase parents’ stress levels. Therefore, policymakers and developers of community-based interventions and prevention initiatives should address problems caused by the living environment to support Asian immigrants’ parenting challenges and mental health status. In addition, we found that neighborhood racial and ethnic diversity (i.e., a higher percentage of non-Asians) was related to lower parenting stress among Asian immigrant families. Traditional Asian cultures heavily emphasize the education of children, and Asian parents are widely believed to have strict parenting styles (e.g., “tiger moms”) and self-sacrifice for their children [13]. Living in a community with a higher percentage non-Asian residents might decrease the resulting peer pressure from this parenting style and residents might become more open-minded as they are exposed to other cultures and parenting beliefs. Such experiences may relieve Asian parents of their high standards toward them and their children and subsequently attenuate their stress [28,61]. This finding also suggests that to further relieve Asian parents of their parenting stress, more support from the same ethnicity should be emphasized in community-level practice for neighborhoods with a higher percentage of Asian residents. Interventions or prevention initiatives to protect Asian immigrants from judgments from other ethnicities and educational and social service systems should also be emphasized. For example, recruiting a diverse body of practitioners in community agencies and hosting more community activities like neighbors’ luncheons, summer pool parties in a community, etc.

Interestingly, no significant relationship was detected between the residential instability of a neighborhood and Asian immigrant parents’ parenting stress. One potential explanation for this finding is that during recent decades, it has become more common for people, especially immigrants, to rent rather than buy a home [62]. As a result, the percentage of renter-occupied households may no longer be a sensitive index to measure the quality of a neighborhood. Additionally, inflation, the development of transportation, and diverse lifestyles influence people’s decisions to move from one place to another more frequently. More people prefer renting rather than buying a house, regardless of their socioeconomic status. That is, residential instability might no longer reflect a neighborhood disadvantage.

### Indirect effects of cultural orientation and social support

Individuals’ behaviors are highly related to their multilevel environment and life experiences [19]. For immigrants, their immigration experiences significantly affect their values, beliefs, and parenting behaviors. In particular, cultural orientation and social support are two of the most important factors in their immigration experiences. This study found that economic disadvantage was positively associated with cultural values and further related to elevated parenting stress among Asian immigrant parents. This means that living in an economically disadvantaged community is related to Asian immigrant parents’ preference to keep their traditional cultural values, further increasing their parenting stress, which is consistent with previous literature related to Asian immigrants’ parenting [47]. Previous empirical research indicated that a stronger preference among Asian immigrants for their traditional cultural beliefs is related to stronger family conflicts and mental pressure [47]. Our finding offers empirical evidence of the effects of neighborhood influences on this relationship. Our study showed that living in an economically disadvantaged community negatively affected Asian immigrants’ acculturation process and made them more likely to follow their original cultural beliefs. Another potential fact is that families living in economically disadvantaged communities may have more limited cultural experiences, so they have fewer opportunities to understand and adopt American culture, which leads to their preference for their traditional culture. Therefore, to promote healthy immigration processes, improving Asian immigrants’ living environment would be a good strategy to relieve their parenting stress from acculturation.

Additionally, we found that social support mediated the association between neighborhood economic disadvantage and parenting stress in Asian immigrant families. Specifically, higher levels of neighborhood economic disadvantage were associated with lower levels of social support, which in turn, predicted higher levels of parenting stress. Our findings are similar to prior research that found a mediating role of social support in the link between neighborhood factors (social cohesion) and parenting stress [7]. Neighborhood disadvantage and its associated characteristics, such as poverty, crime, violence, and conflict, can hinder active socialization among neighbors [63]. That is, individuals living in socioeconomically disadvantaged neighborhoods may have less frequent or stable social contact or interaction with other residents in their neighborhoods due to safety concerns, stress, and frequent moves and thus, have weak social support networks [64,65]. Having a robust social support system is important for parents. Strong social support is linked to less parenting stress and more effective parenting practices [7,64,66]. Parents with more social support tend to feel less anxious and more comfortable about close interpersonal relationships and have more positive parent–child interactions [67]. Similarly, greater social support has been associated with better parenting outcomes, including lower parenting stress, greater parental nurturance, and less child maltreatment, among Asian American immigrant parents [68–70]. Taken together, our findings suggest that social support may be a critical intervention target for reducing parenting stress and promoting positive, effective parenting among Asian American immigrant parents in economically disadvantaged neighborhoods.

As for the indirect influences of ethnic heterogeneity on parenting stress, findings suggest that both cultural values and cultural practice played an important mediating role in this relationship. Consistent with previous research [42,47,48], living in a community with higher ethnic diversity increased Asian immigrants’ preference for their cultural beliefs, which further increased their parenting stress. Living in a neighborhood with more residents from other ethnicities might increase opportunities for Asian immigrants to be positively recognized for their special cultural heritage and identity, but it might also bring more sensitivity about their traditional cultural beliefs. This awareness might also bring more stress due to discrimination or other factors related to their minority identity, according to the minority stress framework [50,51]. Therefore, comprehensively understanding both the living environment and families’ cultural beliefs is extremely important for practitioners. Every Asian immigrant family has unique immigration experiences and lives in communities with different levels of ethnic diversity, which can lead to different preferences toward their culture and American culture. Being aware of ethnic diversity is crucial for community policies and interventions.

Interestingly, compared with cultural values, ethnic heterogeneity showed opposite effects on cultural practices among Asian immigrant parents. Living in a neighborhood with more residents from other ethnicities made Asian immigrants more likely to practice American culture than Asian culture, which increased their parenting stress. Different from cultural values, cultural practice is more about behaviors, like spoken language and daily life patterns, which are more likely to be observed by others. Therefore, it is possible that even though some Asian immigrants prefer their traditional culture, they more frequently engage in Western behaviors due to other reasons, including convenience and social needs. Interventions and prevention initiatives to protect Asian immigrant parents from burning out should pay attention to both their cultural values and practices to make sure that they get enough endorsement and support from practitioners regardless of their cultural orientation.

Furthermore, this study found that ethnic heterogeneity did not significantly affect social support, nor was social support a mediator of the relationship between ethnic heterogeneity and parenting stress in Asian immigrant families. This finding is inconsistent with previous literature related to the general population [7,53]. Because no previous studies investigated the mediating effects of social support on the relationship between ethnic heterogeneity and parenting stress among Asian immigrant parents, the current study provides empirical evidence to show that Asian immigrants’ living environments may not affect their social support system. Neighborhood ethnic heterogeneity played a more important role in affecting Asian immigrants’ cultural orientation, compared with social support. Nowadays, an individual’s social support can come from their daily life or remote families or friends, which significantly benefits immigrants because they have more chances to connect with their families and friends in their home country. However, further investigation is needed to capture the relationship among neighborhood ethnic diversity, social support, and parenting stress among Asian immigrant families.

### Limitations

This study needs to be considered in light of several limitations. First, this cross-sectional study design prevented analysis of causal relationships among neighborhood influences, cultural orientation, social support, and Asian immigrants’ parenting stress. Second, these data were collected in New York, New Jersey, and Hawaii, which limits the generalizability of the study results. Finally, considering the inconsistency of the relationship among neighborhood ethnic diversity, social support, and parenting stress among Asian immigrant families, further empirical studies are needed to provide a comprehensive understanding of this relationship.

### Conclusion

In conclusion, our study examined the mechanism between neighborhood disorganization and Asian immigrant parents’ parenting stress. It is the first study to investigate the interactive effects among neighborhood structural factors, cultural orientation, social support, and parenting stress among Asian immigrants. According to our findings, further research and practice should focus on: (a) community-based practice for Asian immigrant communities to promote mental health and parenting; and (b) factors and inventions that can effectively facilitate Asian immigrant families’ healthy community experiences and cultural orientation.

## Supporting information

S1 File (ZIP)
